# Phase II study and biomarker analysis of cetuximab combined with modified FOLFOX6 in advanced gastric cancer

**DOI:** 10.1038/sj.bjc.6604861

**Published:** 2009-01-06

**Authors:** S-W Han, D-Y Oh, S-A Im, S R Park, K-W Lee, H S Song, N-S Lee, K H Lee, I S Choi, M H Lee, M A Kim, W H Kim, Y-J Bang, T-Y Kim

**Affiliations:** 1 Department of Internal Medicine, Seoul National University Hospital, Seoul, Korea; 2 Cancer Research Institute, Seoul National University College of Medicine, Seoul, Korea; 3 National Cancer Center, Goyang, Korea; 4 Seoul National University Bundang Hospital, Seongnam, Korea; 5 Keimyung University School of Medicine, Daegu, Korea; 6 Soonchunhyang University College of Medicine, Seoul, Korea; 7 Yeungnam University College of Medicine, Daegu, Korea; 8 Seoul Municipal Boramae Hospital, Seoul, Korea; 9 Inha University Hospital, Incheon, Korea; 10 Department of Pathology, Seoul National University College of Medicine, Seoul, Korea

**Keywords:** Cetuximab, chemotherapy, epidermal growth factor, epidermal growth factor receptor, gastric cancer, transforming growth factor-*α*

## Abstract

This prospective study was conducted with the Korean Cancer Study Group to evaluate the efficacy and safety of cetuximab combined with modified FOLFOX6 (mFOLFOX6) as first-line treatment in recurrent or metastatic gastric cancer and to identify potential predictive biomarkers. Patients received cetuximab 400 mg m^−2^ at week 1 and 250 mg m^−2^ weekly thereafter until disease progression. Oxaliplatin (100 mg m^−2^) and leucovorin (100 mg m^−2^) were administered as a 2-h infusion followed by a 46-h continuous infusion of 5-fluorouracil (2400 mg m^−2^) every 2 weeks for a maximum of 12 cycles. Biomarkers potentially associated with efficacy were analysed. Among 38 evaluable patients, confirmed response rate (RR) was 50.0% (95% CI 34.1–65.9). Median time-to-progression (TTP) was 5.5 months (95% CI 4.5–6.5) and overall survival (OS) 9.9 months. Eleven patients having tumour EGFR expression by immunohistochemistry with low serum EGF and TGF-*α* levels showed a 100% RR compared to 37.0% in the remaining 27 patients (*P*<0.001). Moreover, ligand level increased when disease progressed in seven out of eight patients with EGFR expression and low baseline ligand level. No patient exhibited EGFR amplification or K-ras mutations. Gastric cancer patients with EGFR expression and low ligand levels had better outcomes with cetuximab/mFOLFOX6 treatment.

Gastric cancer is one of the most common cancers worldwide with a particularly high incidence in Asian countries including Korea (Shin *et al*, 2004; Kamangar *et al*, 2006). Regardless of geographical incidence, gastric cancer is also associated with high mortality as many patients present with locally advanced or metastatic disease, and treatment options are limited (Kamangar *et al*, 2006). Thus, it is one of the more therapeutically challenging cancers for oncologists. Although recent efforts to improve the treatment of gastric cancer have shown positive results, the outcome of advanced disease is still disappointing (Van Cutsem *et al*, 2006; Cunningham *et al*, 2008). Targeted agents are therefore being investigated in an effort to improve survival of gastric cancer patients (Tabernero *et al*, 2005).

Cetuximab (Erbitux; Merck KGaA, Darmstadt, Germany) is an immunoglobulin G1 monoclonal antibody that binds the epidermal growth factor receptor (EGFR) with high affinity, which competitively blocks ligand binding, inhibits tyrosine kinase activation and results in receptor downregulation (Mendelsohn and Baselga, 2003). Cetuximab has shown promising results in EGFR-expressing metastatic colorectal cancer in both the first-line setting and in patients with refractory disease (Cunningham *et al*, 2004; Jonker *et al*, 2007; Van Cutsem *et al*, 2007). Cetuximab plus chemotherapy (irinotecan/5-FU/leucovorin (FOLFIRI) and weekly oxaliplatin/5-FU/leucovorin (FUFOX)) has also shown favourable results as a first-line treatment of advanced gastric or gastroesophageal junction adenocarcinoma in phase II studies (Lordick *et al*, 2007; Pinto *et al*, 2007).

Recent biomarker studies suggest that selection of patients according to their biomarker status may improve treatment outcomes. Biomarker analysis of cetuximab in colorectal cancer has identified potential biomarkers such as EGFR gene amplification and K-ras mutations that may guide treatment decisions (Moroni *et al*, 2005; Khambata-Ford *et al*, 2007; Sartore-Bianchi *et al*, 2007; Zhang *et al*, 2007; Bokemeyer *et al*, 2008; Cappuzzo *et al*, 2008; Lievre *et al*, 2008; Van Cutsem *et al*, 2008; Tejpar *et al*, 2008b). The additional activity of cetuximab combined with chemotherapy was limited to K-ras wild-type colorectal cancers (Bokemeyer *et al*, 2008; Van Cutsem *et al*, 2008). Other potential biomarkers of cetuximab activity in colorectal cancer include EGFR ligands (epiregulin and amphiregulin) and polymorphisms in EGFR, EGF, and Fc fragment of IgG receptor (Khambata-Ford *et al*, 2007; Zhang *et al*, 2007; Graziano *et al*, 2008; Tejpar *et al*, 2008a). However, there is only limited data regarding biomarkers in gastric cancer patients treated with cetuximab (Rojas Llimpe *et al*, 2007).

This study evaluated cetuximab in combination with modified FOLFOX6 (mFOLFOX6), which is one of the most widely used first-line chemotherapy regimens in Korea, in previously untreated patients with advanced gastric cancer. Moreover, to identify candidate biomarkers for optimal patient selection of cetuximab/mFOLFOX6 treatment in gastric cancer, we have investigated biomarkers that may be associated with efficacy.

## Patients and methods

### Study design

This study was a prospective multicentre phase II study performed in eight centres of the Korean Cancer Study Group (KCSG). The primary end point was overall response rate according to the Response Evaluation Criteria in Solid Tumours (RECIST) (Therasse *et al*, 2000). The secondary end points included time-to-progression, overall survival, toxicity, and pharmacogenomic analysis. Written informed consent was received prior to study entry. The study protocol was reviewed and approved by the Institutional Review Boards at the participating institutions. Recommendations of the Declaration of Helsinki for biomedical research involving human subjects were also followed.

### Patients and treatments

The main inclusion criteria were age ⩾18 years, Eastern Cooperative Oncology Group (ECOG) performance status (PS) ⩽2, histologically confirmed adenocarcinoma of the stomach, recurrent or metastatic disease, no prior chemotherapy, radiotherapy, immunotherapy, or EGFR pathway-targeting therapy (prior adjuvant chemotherapy completed >12 months from the study medication was permitted), adequate bone marrow, hepatic, and renal function, and at least one measurable lesion. Exclusion criteria included intestinal obstruction or impending obstruction, active tumour bleeding, interstitial pneumonitis or symptomatic pulmonary fibrosis, pregnant or breastfeeding patients, other serious diseases, and peripheral neuropathy of grade 1.

Patients received an initial dose of cetuximab 400 mg m^−2^ followed by weekly doses of 250 mg m^−2^. Modified FOLFOX6 comprised of oxaliplatin 100 mg m^−2^ and leucovorin 100 mg m^−2^ given intravenously over 2 h on day 1 followed by a 46 h infusion of 5-fluorouracil (5-FU) 2400 mg m^−2^, which was repeated every 2 weeks. Patients received a maximum of 12 cycles of mFOLFOX6. Cetuximab was continued as a monotherapy until disease progression.

Response evaluation was performed following the RECIST criteria (Therasse *et al*, 2000). Computed tomography (CT) scans were performed every 6 weeks during the cetuximab/mFOLFOX6 treatment period and every 8 weeks afterwards. Complete or partial responses were confirmed with CT scans taken at least 4 weeks apart. Adverse events were assessed using National Cancer Institute Common Terminology Criteria for Adverse Events version 3.0.

### Biomarker analysis

Biomarker analysis of tumour was performed with formalin-fixed paraffin-embedded tissue blocks. In relapsed cases, tissue specimen obtained after recurrence was required for the study entry. However, in two cases found to have inadequate tumour left in these blocks, tissue from prior gastrectomy was used. Tissue of origin was stomach in 34, liver in three, and abdominal soft tissue in one. Immunohistochemistry (IHC) of thymidylate synthase, thymidine phosphorylase (TP), and excision repair cross-complementation group 1 (ERCC1) was performed as described in Supplementary Table 1. Expression of EGFR and HER2 was determined using the PharmDx kit (DAKO, Carpinteria, CA, USA) and the HercepTest kit (DAKO), respectively, according to the manufacturer's instructions. Staining was carried out using an Autostainer 360 (Lab Vision, Fremont, CA, USA). For quantitative scoring of IHC, an IHC score (0–300) was derived by multiplying the staining intensity (0, 1, 2, 3) by the percentage of positive cells (0–100). Gene amplifications of EGFR and HER2 were detected by fluorescence *in situ* hybridization (FISH) using LSI EGFR/CEP 7 Dual Color Probe (Vysis, Des Plaines, IL, USA) for EGFR and PathVysion (Vysis) for HER2 following the manufacturer's instructions. Blinded scoring of IHC and FISH was performed by two pathologists (MAK and WHK). For the mutational analysis, only the areas in which cancer cells occupied more than 60% of the total area assessed by H&E slide review were selected for DNA extraction. Direct sequencing of nested polymerase chain reaction (PCR) products of K-ras exons 1 and 2 was performed using primers listed in Supplementary Table 2. Enzyme-linked immunosorbent assay (ELISA) of serum samples acquired before treatment and at the time of disease progression was performed using commercially available kits following the manufacturer's instructions for the following markers: EGFR extracellular domain (Calbiochem, San Diego, CA, USA), EGF (R&D Systems, Minneapolis, MN, USA), TGF-*α* (R&D Systems), and amphiregulin (R&D Systems). Samples were assayed in duplicate.

### Statistical analysis

This study was designed to test the hypothesis that the response rate of the study treatment would be 70% (H_1_), which is significantly different from 40% (H_0_). The H_0_ and H_1_ values were demanded by the Korean Food and Drug Administration for approval of the study. Sample size was determined following Simon 2-stage design with a type I and II error of 5% each (Simon, 1989). Fourteen patients were enrolled in the first stage. When six or more responses were observed, the second stage was initiated to enroll 20 additional patients for a total of 34 patients. To reject H_0_, 19 responses were required among 34 patients. Assuming a 15% dropout rate, the total number of patients needed for the study was 40.

For the selection of a cutoff point for the IHC score and ligand level, a receiver operating characteristic curve analysis was utilised in which the IHC score was also regarded as a continuous variable. The IHC score and ligand level with the highest sensitivity and specificity for response was chosen as the cutoff. Statistical analysis of biomarker status and response rate was carried out using Pearson's *χ*^2^ test or Fisher's exact test. Serum ligand levels were compared using the Kruskal–Wallis test or Mann–Whitney *U*-test. Multivariate analysis of response was performed with the backward stepwise logistic regression model. Median durations of TTP and OS were calculated using the Kaplan–Meier method. Comparisons of TTP and OS were made with log-rank tests. Multivariate analysis of TTP and OS were carried out using the backward stepwise Cox regression model. In the multivariate analysis, biomarkers with *P*<0.20 were included as covariates. To adjust for baseline characteristics, sex, age, ECOG PS (0 *vs* 1–2), Lauren classification, and additional characteristics with *P*<0.20 (site and number of involved organs) were also included. Two sided *P*-values of less than 0.05 were considered significant. All analyses were performed using SPSS for Windows, version 12.0 (SPSS Inc., Chicago, IL, USA).

## Results

### Patients

Between December 2006 and June 2007, 40 patients with recurrent or metastatic gastric cancer were enrolled into the study. Baseline patient and disease characteristics are presented in Table 1. Of the 40 patients enrolled, one patient withdrew his consent and refused any further follow-up immediately following completion of 1st cycle of mFOLFOX6 treatment and another patient developed gastric perforation 1 week after treatment initiation the cause of which was unclear. These two patients are excluded from the efficacy analysis and the former patient is also excluded from the safety analysis. Therefore, 38 patients were evaluable for response and 39 patients assessable for safety. At the time of data cutoff at the end of January 2008, four patients were still receiving cetuximab maintenance monotherapy. Median dose intensities of cetuximab, oxaliplatin, and 5-fluorouracil were 100% (range 66.6–100%), 92.3% (48.0–100%), and 92.2% (55.4–100%), respectively.

### Efficacy and safety

Among the 38 evaluable patients, the best overall response was partial response (PR) in 19 patients (50.0%, 95% confidence interval (CI) 34.1–65.9). All PRs were confirmed after 4 weeks. Stable disease (SD) was observed in 16 patients (42.1%, 95% CI 26.4–57.8), and progressive disease (PD) in three patients (7.9%, 95% CI 0–16.5). The disease control rate (PR + SD) was 92.1% (95% CI 83.5–100). In the intent-to-treat analysis, response rate and disease control rate were 47.5% (95% CI 32.0–63.0) and 87.5% (95% CI 77.3–97.7), respectively. Among the 16 patients with SD, there were two unconfirmed responders (one withdrawal of consent after obtaining a PR, and one death unrelated to disease or treatment prior to confirmation of the response). These two unconfirmed responders are included in the responder group in the following biomarker analysis. Median time-to-progression was 5.5 months (95% CI: 4.5–6.5) (Supplementary Figure 1A). Until January 2008 (median duration of follow-up 9.8 months), 18 death events occurred among 38 evaluable patients. Median overall survival was 9.9 months (Supplementary Figure 1B).

Cetuximab in combination with mFOLFOX6 was generally well-tolerated with Grade ⩾3 adverse events reported as expected for this treatment combination. Fifteen patients (38.5%) experienced any kind of grade 3 or 4 adverse event. The most commonly reported Grade 3/4 adverse events were neutropenia, diarrhoea, stomatitis, and rash (Supplementary Table 3). One adverse event related to treatment (febrile neutropenia) led to death.

Patients who did not develop skin rash (five patients) had shorter TTP (*P*=0.011, median 1.3 *vs* 5.6 months) and OS (*P*=0.008, median 2.4 months *vs* not reached) compared to the patients who developed any grade of skin rash (33 patients). Response rates were 20.0 and 54.5%, respectively (*P*=0.34). No significant difference in TTP or OS was seen between the grades of rash.

### Biomarker analyses

**Figure 1:**
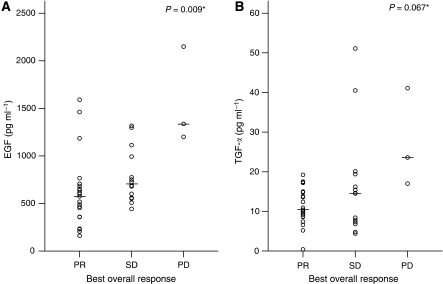
Baseline serum EGF (**A**) and TGF-*α* (**B**) levels according to the best overall response. Bars indicate median values. *P*-value by Kruskal–Wallis test. Abbreviations: EGF, epidermal growth factor; TGF, transforming growth factor; PR, partial response; SD, stable disease; PD, progressive disease.

Among the biomarkers tested, low serum levels of EGF (<667 pg ml^−1^) and TGF-*α* (<14 pg ml^−1^) were significantly associated with a higher response rate (Table 2). Serum EGF level was significantly different according to best overall response and TGF-*α* level showed a similar trend (Figure 1). In the multivariate analysis, low serum EGF level was significantly associated with response (adjusted HR 11.8, 95% CI 1.8–75.4; *P*=0.009). No marker was significantly associated with TTP in the univariate analysis (Table 2). Nevertheless, EGFR expression in the tumour was significantly associated with longer TTP in the multivariate analysis (adjusted HR 0.23, 95% CI 0.086–0.63; *P*=0.004). In the univariate analysis of OS, low TP and ERCC1 expression in the tumour was associated with longer OS (Table 2). In the multivariate analysis, only a low tumour TP expression was associated with longer survival (adjusted HR 0.26, 95% CI 0.097–0.70; *P*=0.008).

**Figure 2:**
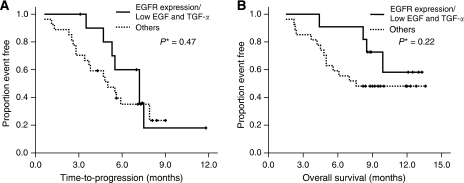
Kaplan–Meier curves of time-to-progression (**A**) and overall survival (**B**) according to EGFR expression and serum ligand status. *P*-value by log-rank test. Abbreviations: EGFR, epidermal growth factor receptor; EGF, epidermal growth factor; TGF, transforming growth factor.

**Figure 3:**
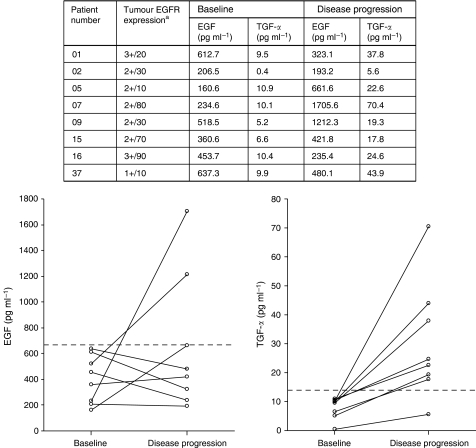
Serum EGF and TGF-*α* level at baseline and disease progression in patients with tumour EGFR expression and low initial ligand levels. ^a^Staining intensity/percentage of positive cells. Dotted lines in the figures represent cutoff values of each ligand (EGF 667 pg ml^−1^ and TGF-*α* 14 pg ml^−1^). Abbreviations: EGFR, epidermal growth factor receptor; EGF, epidermal growth factor; TGF, transforming growth factor.

To identify a patient subgroup that is most likely to benefit from the treatment, combinations of biomarkers were evaluated. Interestingly, all of the patients (*N*=11) having EGFR tumour expression detected by immunohistochemistry together with low levels of both serum EGF (<667 pg ml^−1^) and TGF-*α* (<14 pg ml^−1^) showed a response. Response rate in the remaining patients (*N*=27) was 37.0% (*P*<0.001). Serum EGF and TGF-*α* levels were lower in responders with EGFR expression compared to non-responders, whereas no association between serum ligand level and response was found in patients with negative EGFR expression (Supplementary Figure 2). TTP (*P*=0.47; median 7.2 *vs* 5.0 months, respectively) and OS (*P*=0.22; not reached *vs* 7.6 months, respectively) were not significantly different in the univariate analysis (Figure 2). Nonetheless, after adjusting for clinical factors (age, sex, PS, Lauren classification, site and number of involved organs), TTP (adjusted HR 0.28, 95% CI 0.09–0.82; *P*=0.020) and OS (adjusted HR 0.16, 95% CI 0.04–0.68; *P*=0.013) were also significantly longer in these patients with EGFR expression and low levels of ligands. Moreover, among eight patients with follow-up serum samples collected at the time of disease progression, seven patients showed elevation of EGF or TGF-*α* level above the cutoff values (Figure 3).

Although recent studies have shown a significant association between the response to cetuximab treatment and EGFR amplification or K-ras mutations in colorectal cancer, none of the patients in the present study exhibited increased EGFR gene copy number (EGFR/CEP7 >2.0) or K-ras mutations.

## Discussion

Targeting EGFR with monoclonal antibodies or tyrosine kinase inhibitors has improved treatment strategies against cancer during the past few years. Patient selection strategy for these agents is an important issue. Randomised studies of cetuximab combined with chemotherapy in the first-line treatment of colorectal cancer clearly showed that the benefit of adding cetuximab was limited to K-ras wild-type cancers (Bokemeyer *et al*, 2008; Van Cutsem *et al*, 2008). In contrast, the addition of cetuximab to FOLFOX-4 had a detrimental effect on response and progression-free survival in K-ras mutant tumours in the OPUS trial (Bokemeyer *et al*, 2008). High EGFR gene copy number has been associated with better response to cetuximab or panitumumab (Moroni *et al*, 2005; Sartore-Bianchi *et al*, 2007; Cappuzzo *et al*, 2008). However, no patient had K-ras mutation or increased EGFR gene copy number in this study. Previous studies performed in large number of patients also demonstrate that K-ras mutation or increased EGFR gene copy number is an uncommon genetic event in gastric cancer (Lee *et al*, 2003; Kim *et al*, 2008). These data suggest that a biomarker application needs to be based on the specific type of cancer.

In this study, we have failed to show a pre-specified improvement of response rate by addition of cetuximab to mFOLFOX6. This may be due to the unfavourable baseline characteristics of the patients: more than half of the patients enrolled had peritoneal seeding and three or more involved organs even though most of the patients had good PS. Another reason for the failure could be the H1 set too high. In fact, the response rate and disease control rate in this study is similar to those from cetuximab plus FOLFIRI (44.1 and 91.2%, respectively) (Pinto *et al*, 2007). However, TTP and OS are inferior to cetuximab plus FOLFIRI (Pinto *et al*, 2007). In comparison with the recent three-drug combination chemotherapies, cetuximab plus mFOLFOX6 showed no better results in terms of efficacy but with less toxicity, especially neutropenia (Van Cutsem *et al*, 2006; Cunningham *et al*, 2008).

More importantly, the failure of improvement may be due to a differential effect on the response rate from the addition of cetuximab according to the molecular status of the tumour, as seen with K-ras mutation and cetuximab plus FOLFOX in colorectal cancer (Bokemeyer *et al*, 2008). Therefore, it is important to identify who could benefit from cetuximab and who may be potentially harmed by it in gastric cancer.

In the biomarker analysis, we have identified a subgroup of patients that shows a more favourable outcome of who could be the patients benefiting from cetuximab. Patients having EGFR expression and low levels of the major ligands, EGF and TGF-*α*, showed a 100% response rate. Considering the fact that cetuximab is a monoclonal antibody that specifically targets EGFR and competitively inhibits ligand binding, it is not unexpected that the patients with tumour expression of EGFR and low levels of competitive ligands showed better treatment outcome (Mendelsohn and Baselga, 2003). Elevation of ligand levels at the time of disease progression further supports the important role of these ligands in resistance to cetuximab treatment. High levels of serum EGFR ligands have also been implicated in resistance to gefitinib in lung cancer (Ishikawa *et al*, 2005). However, in colorectal cancer patients receiving cetuximab, high gene expression of epiregulin and amphiregulin in tumour was associated with better outcome (Khambata-Ford *et al*, 2007; Tejpar *et al*, 2008a). It is likely that different ligands may have distinct interactions with the various EGFR targeting agents. It is also possible that different ligands may have different roles within autocrine EGFR activation loop in different cancers. As this study was a single arm phase II study, whether the better outcome of the patients is a result of the incorporation of cetuximab into the treatment, or is a consequence of a possible innate good prognosis should be investigated in future randomised studies.

There are limited but controversial reports regarding the prognostic implication of EGFR expression in gastric cancer (Gamboa-Dominguez *et al*, 2004; Lieto *et al*, 2008; Matsubara *et al*, 2008). In a recent study of cetuximab in gastric cancer which also included EGFR-negative tumours, EGFR expression was not associated with the response rate (Lordick *et al*, 2007). In contrast, EGFR expression was an independent predictor of longer TTP in the multivariate analysis in this study. Moreover, combined analysis with serum ligand status further improved the selection of patients having better outcomes. HER2 positive rate by IHC (15.8%) or FISH (13.2%) is similar to previous reports considering that all patients had gastric cancer and majority of patients had diffuse type in the present study (Tanner *et al*, 2005; Leon-Chong *et al*, 2007). In contrast to previous reports showing poor survival of HER2-positive gastric cancer patients who underwent surgery, HER2-positive patients showed a non-significant trend towards a better outcome in this study (Allgayer *et al*, 2000; Tanner *et al*, 2005). HER2 may have a predictive role in cetuximab treatment of gastric cancer. Low TP expression having association with better OS is in accordance with previous studies which were performed in gastrointestinal cancer patients who received 5-FU-based treatments (Metzger *et al*, 1998; Napieralski *et al*, 2005).

The main limitation of this study was the sample size, which was not large enough to test the differences between the statuses of various biomarkers. Despite this, biomarkers were identified which are independently associated with response or survival, which we believe merit further investigation in randomised studies.

In conclusion, cetuximab in combination with mFOLFOX6 as a first-line treatment in gastric cancer showed the most promising results in patients with EGFR expression and low serum ligand levels (EGF and TGF-*α*). This combination treatment in gastric cancer warrants further evaluation in a large-scale study with biomarker analysis, including EGFR and ligand status, for future optimisation of patient selection.

## Figures and Tables

**Table 1:** Patient and disease characteristics at baseline

**Characteristic**	**Number of patients (*N*=40)**	**%**
*Sex*		
Male	30	75.0
Female	10	25.0
		
*Age, years*		
Median	55	
Range	33–74	
		
*Performance status (ECOG)*		
0	7	17.5
1	29	72.5
2	4	10.0
		
*Disease status*		
Relapsed	5	12.5
Adjuvant chemotherapy	3	
No adjuvant chemotherapy	2	
Initially metastatic	35	87.5
		
*Location*		
Proximal	5	12.5
Middle	4	10.0
Distal	26	65.0
Diffuse	5	12.5
		
*Lauren classification*		
Intestinal	12	30.0
Diffuse	28	70.0
		
*Number of organs involved*		
1	5	12.5
2	7	17.5
3	17	42.5
4	7	17.5
⩾5	4	10.0
		
*Site of metastasis*		
Lymph node	36	90.0
Peritoneum	21	52.5
Liver	16	40.0
Others (lung, bone, and so on)	9	22.5

ECOG, Eastern Cooperative Oncology Group.

**Table 2:** Univariate analyses of biomarker and treatment outcomes

	**Criteria (No. of patients)**	**Responders (%)**	***P*-value**	**Median TTP (mo)**	***P*-value**	**Median OS (mo)**	***P*-value**
*Tumour expression (IHC)*							
TS^a^	<25 (13)	9 (69.2)	0.21	4.8	0.78	N/R	0.15
	>25 (25)	12 (48)		5.5		8.2	
TP^a^	<25 (20)	13 (65)	0.20	7.2	0.10	N/R	0.021
	>25 (18)	8 (44.4)		3.8		7.0	
ERCC1^a^	<130 (16)	11 (68.8)	0.15	7.2	0.60	N/R	0.022
	>130 (22)	10 (45.5)		5.0		7.0	
EGFR^b^	(−) (12)	5 (41.7)	0.25	2.8	0.18	6.1	0.33
	1+ in ⩾10% (26)	16 (61.5)		5.9		N/R	
HER2^b^	0/1+ (32)	16 (50)	0.20	5.3	0.20	9.9	0.47
	2+/3+ (6)	5 (83.3)		7.2		N/R	
							
*Gene copy number (FISH)*							
HER2/CEP17	<2.0 (33)	18 (54.5)	1.0	5.3	0.18	9.9	0.66
	>2.0 (5)	3 (60)		N/R		N/R	
							
*Serum protein level (ELISA)*							
EGFR (ng ml^−1^)	<41.9 (13)	6 (46.2)	0.42	5.5	0.73	5.6	0.12
	>41.9 (25)	15 (60)		5.5		N/R	
EGF (pg ml^−1^)	<667 (20)	15 (75)	0.01	7.6	0.88	N/R	0.51
	>667 (18)	6 (33.3)		5.0		7.6	
TGF-*α* (pg ml^−1^)	<14 (21)	15 (71.4)	0.03	5.9	0.47	N/R	0.31
	>14 (17)	6 (35.3)		4.8		7.6	
Amphiregulin (pg ml^−1^)	<1.14 (16)	7 (43.8)	0.22	5.0	0.89	6.1	0.34
	>1.14 (22)	14 (63.6)		7.2		N/R	

CEP, chromosome enumerator probe; EGF, epidermal growth factor; EGFR, epidermal growth factor receptor; ELISA, enzyme-linked immunosorbent assay; ERCC1, excision repair cross-complementation group 1; FISH, fluorescence *in situ* hybridization; HER2, human epidermal growth factor receptor 2; IHC, immunohistochemistry; mo, months; N/R, not reached; OS, overall survival; TGF, transforming growth factor; TP, thymidine phosphorylase; TS, thymidylate synthase; TTP, time-to-progression.

a Numbers in the criteria denote IHC scores derived from staining intensity and percentage of positive cells.

b IHC score cutoff for EGFR was 7.5 which was identical to 1+ staining in 10% or more cancer cells. The cutoff for HER2 was 15, which was identical to 2+ staining in at least 10% of cells.
