# Supplementary material for: Phase II study and biomarker analysis of cetuximab combined with modified FOLFOX6 in advanced gastric cancer
Source: Br J Cancer. 2009 Jan 6;100(2):298–304. doi: 10.1038/sj.bjc.6604861 (PMC2634707; doi:10.1038/sj.bjc.6604861)
Supplement: Supplementary Figures 1 and 2 [file 6604861x1.ppt]

## Slide 1
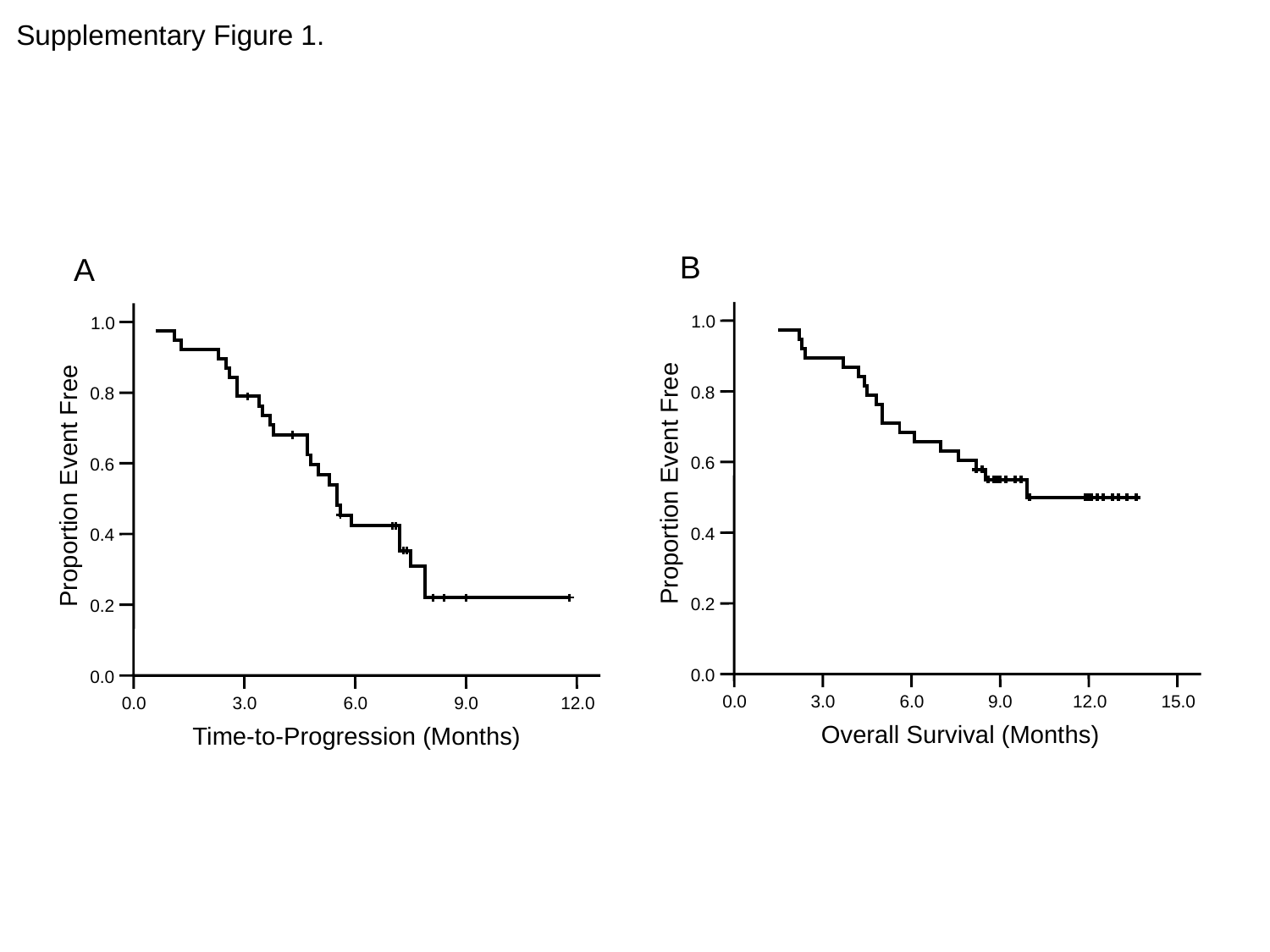

Supplementary Figure 1.
B
A
1.0
1.0
0.8
0.8
0.6
0.6
Proportion Event Free
Proportion Event Free
0.4
0.4
0.2
0.2
0.0
0.0
0.0
3.0
6.0
9.0
12.0
15.0
0.0
3.0
6.0
9.0
12.0
Overall Survival (Months)
Time-to-Progression (Months)

## Slide 2
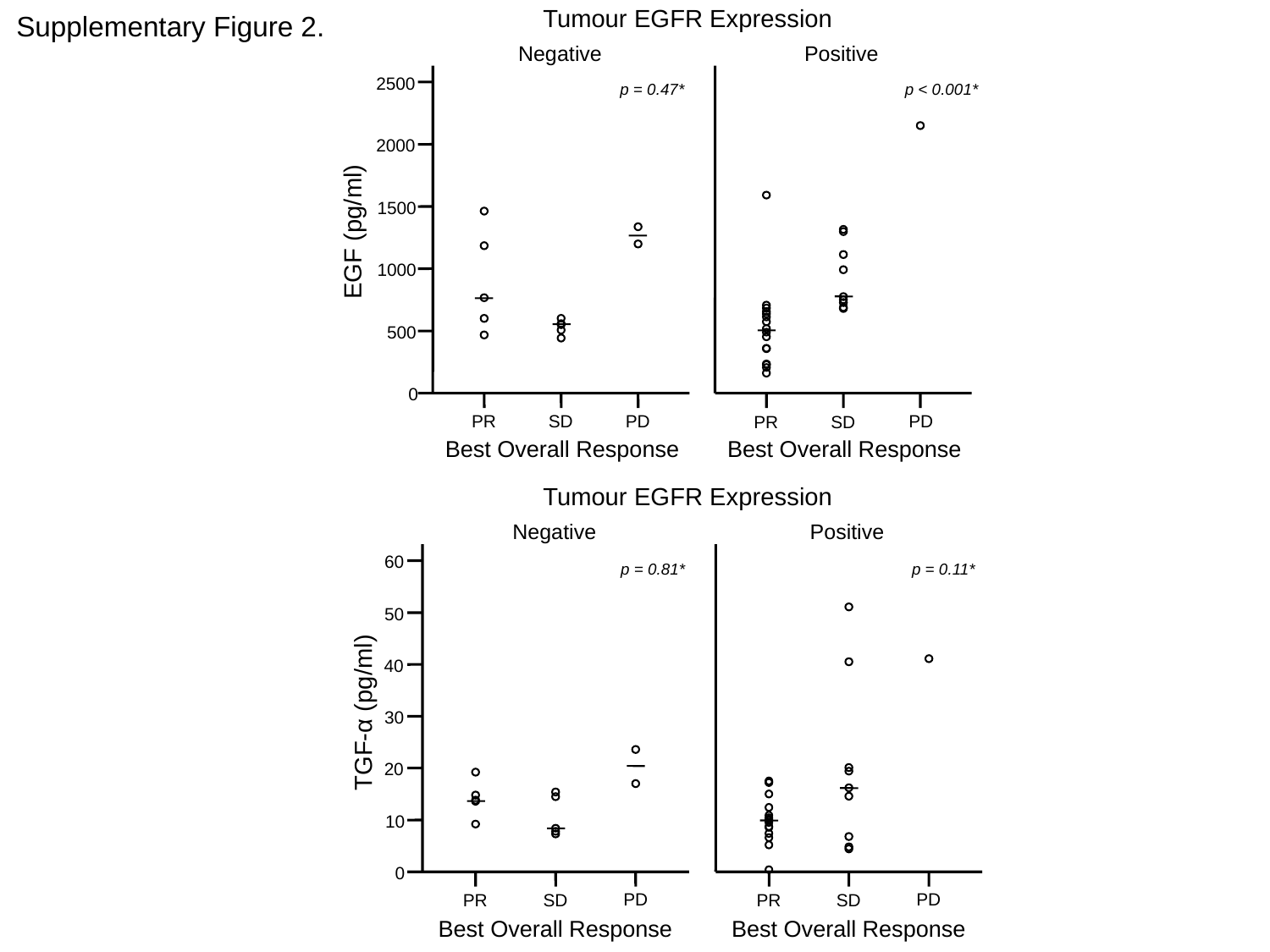

Supplementary Figure 2.
Tumour EGFR Expression
Negative
Positive
2500
p = 0.47*
p < 0.001*
2000
1500
EGF (pg/ml)
1000
500
0
PD
PR
SD
PD
PR
SD
Best Overall Response
Best Overall Response
Tumour EGFR Expression
Negative
Positive
60
p = 0.11*
p = 0.81*
50
40
TGF-α (pg/ml)
30
20
10
0
PD
PD
PR
SD
PR
SD
Best Overall Response
Best Overall Response
