# Supplementary material for: Phase II study and biomarker analysis of cetuximab combined with modified FOLFOX6 in advanced gastric cancer
Source: Br J Cancer. 2009 Jan 6;100(2):298–304. doi: 10.1038/sj.bjc.6604861 (PMC2634707; doi:10.1038/sj.bjc.6604861)
Supplement: Supplementary Tables 1–3 [file 6604861x2.doc]

**Supplementary Table 1.** **Antibodies for immunohistochemistry**

| **Protein** | **Antigen Retrieval Method** | **Primary Antibody** | | | | **Secondary Antibody Kit** |
| --- | --- | --- | --- | --- | --- | --- |
|  |  | **Antibody Clone** | **Manufacturer** | **Dilution** | **Incubation Time and Temperature** |  |
| Thymidylate Synthase (TS) | Microwave incubation in citrate buffer (pH 6.0) for 15 minutes | Mouse monoclonal Ab (TS 106) | Neomarkers (Fremont, CA) | 1:100 | 60 minutes at room temperature | UltraVision LP System (Lab Vision, Fremont, CA) |
| Thymidine Phosphorylase (TP) |  | Mouse monoclonal Ab (P-GF.44C) |  | 1: 40 |  | Histostain Bulk Kit (Invitrogen, Carlsbad, CA) |
| Excision Repair Cross-Complementation Group 1 (ERCC1) |  | Mouse monoclonal Ab (8F1) |  | 1: 100 |  | UltraVision LP System |

**Supplementary Table 2.** **Primer sequences**

| **Gene** |  | **Primers (5’ - 3’)** |  |
| --- | --- | --- | --- |
| **K-ras** | Exon 1 | F1: ggtggagtatttgatagtgtattaacc  R1: tcatgaaaatggtcagagaaacc  F2: tcattatttttattataaggcctgctg  R2: tttatctgtatcaaagaatggtcctg |  |
|  | Exon 2 | F1: ggtgcttagtggccatttgt  R1: cactgctctaatcccccaag  F2: ttcaagtcctttgcccattt  R2: tgcatggcattagcaaagac |  |

**Supplementary Table 3. Adverse events (N = 39)**

| **Adverse event** | **Number of patients (%)** | | | |
| --- | --- | --- | --- | --- |
|  | **Grade 1** | **Grade 2** | **Grade 3** | **Grade 4** |
| **Leucopenia** | 8 (21) | 4 (10) | 3 (8) | 0 |
| **Neutropenia** | 9 (23) | 5 (13) | 4 (10) | 3 (8) |
| **Anaemia** | 10 (26) | 0 | 0 | 0 |
| **Thrombocytopenia** | 11 (28) | 1 (3) | 0 | 1 (3) |
| **AST/ALT elevation** | 22 (56) | 5 (13) | 0 | 0 |
| **Nausea** | 16 (41) | 18 (46) | 1 (3) | 0 |
| **Vomiting** | 10 (26) | 7 (18) | 1 (3) | 0 |
| **Stomatitis** | 10 (26) | 14 (36) | 3 (8) | 0 |
| **Diarrhoea** | 14 (36) | 7 (18) | 4 (10) | 0 |
| **Rash** | 13 (33) | 17 (44) | 3 (8) | 0 |
| **Sensory neuropathy** | 21 (54) | 8 (21) | 1 (3) | 0 |
| **Allergic reaction** | 1 (3) | 6 (15) | 1 (3) | 0 |
| **Pneumonitis** | 3 (8) | 1 (3) | 0 | 0 |

Abbreviations: AST, aspartate amino transferase; ALT, alanine amino transferase.
