# Supplementary material for: Phase II study and biomarker analysis of cetuximab combined with modified FOLFOX6 in advanced gastric cancer
Source: Br J Cancer. 2009 Jan 6;100(2):298–304. doi: 10.1038/sj.bjc.6604861 (PMC2634707; doi:10.1038/sj.bjc.6604861)
Supplement: Supplementary Figure Legends [file 6604861x3.doc]

**Supplementary Figure 1. Kaplan-Meier curves of time-to-progression (A) and overall survival (B) (N = 38)**

**Supplementary Figure 2. Baseline serum ligand level and best overall response according to tumour EGFR expression status**

Bars indicate median values.

* *p*-value by Mann-Whitney U test (PR *vs.* SD/PD).

Abbreviations: EGFR, epidermal growth factor receptor; EGF, epidermal growth factor; TGF, transforming growth factor; PR, partial response; SD, stable disease; PD, progressive disease
